# Engineering chemokines to develop putative anti HIV-1 agents

**DOI:** 10.1186/1471-2334-12-S1-O1

**Published:** 2012-05-04

**Authors:** A Deepu, A Aravind, K Santhosh Kumar

**Affiliations:** 1 Chemical Biology Lab, Rajiv Gandhi Centre for Biotechnology, Thiruvananthapuram, India

## Background

Even though, Highly Active Antiretroviral Therapy (HAART) has resulted in significant reduction in mortality associated with Acquired Immune Deficiency Syndrome (AIDS), the side-effects and difficulties in patient compliance warrants the search for new therapeutic options. One potential strategy is to design chemokine analogues that will prevent the entry of human immunodeficiency virus-1 (HIV-1) into the target cell by competitively blocking its interaction with CC chemokine-5 (CCR5) receptor. The objective of the present study is to design and validate the efficacy of chemokine analogues based on the viral macrophage inflammatory protein-II (vMIP-II) core as putative anti-HIV agents.

## Methods

In the present study we have synthesized the chemokine analogues by Fmoc solid phase peptide synthesis and were purified to homogeneity by semi preparative RP-HPLC. Molecular mass of the analogues were confirmed by MALDI-TOF MS. Structural characterization was done by CD spectroscopy. Their interaction with CCR5 receptor was analyzed by calcium release studies.

## Results

The analogues designed were obtained in high purity and correct identity and they displayed similar patterns on the CD spectra to their parent template vMIP-II. The analogues displayed significantly lower toxicity in MTT assays. The interactive nature of the analogues was evident in calcium release studies where, the calcium flux varies with the type of the grafted N-terminus.

## Conclusion

The findings in this study highlight the potential of vMIP-II to serve as a valuable target in designing chemokine analogues, which have the efficiency to serve as putative anti HIV- 1 agents.

